# Determining the SARS-CoV-2 Anti-Spike Cutoff Level Denoting Neutralizing Activity Using Two Commercial Kits

**DOI:** 10.3390/vaccines10111952

**Published:** 2022-11-18

**Authors:** Engy Mohamed El-Ghitany, Mona H. Hashish, Azza Galal Farghaly, Eman A. Omran

**Affiliations:** 1 Department of Tropical Health, High Institute of Public Health, Alexandria University, Alexandria 21526, Egypt; 2 Department of Microbiology, High Institute of Public Health, Alexandria University, Alexandria 21526, Egypt

**Keywords:** COVID-19, neutralizing antibodies, spike antibodies, receiver operating characteristic (ROC) curve, cutoff point

## Abstract

Background: The viral neutralization assay is the gold standard to estimate the level of immunity against SARS-CoV-2. This study analyzes the correlation between the quantitative Anti-SARS-CoV-2 QuantiVac ELISA (IgG) and the NeutraLISA neutralization assay. Methods: 650 serum samples were tested for both SARS-CoV-2 anti-spike (anti-S) immunoglobulin G (IgG) and neutralizing antibodies (nAbs) using kits by EUROIMMUN, Germany. Results: There was a significant correlation between levels of anti-S and nAbs (Spearman’s rho = 0.913). Among the positive samples for anti-S, 77.0% (n = 345) were positive for nAbs. There was a substantial agreement between anti-S and nAbs (Cohen’s kappa coefficient = 0.658; agreement of 83.38%). Considering NeutraLISA as a gold standard, anti-S had a sensitivity of 98.57%, specificity of 65.66%, NPV of 97.5%, and PPV of 77.0%. When the anti-S titer was greater than 18.1 RU/mL (57.9 BAU/mL), nAbs were positive, with a sensitivity of 90.0% and specificity of 91%. Conclusions: A titer of SARS-CoV-2 anti-S IgG can be correlated with levels of nAbs.

## 1. Introduction

The SARS-CoV-2 genome encodes the spike (S), nucleocapsid, membrane, and envelope structural proteins. The S protein plays a key role in viral infection and pathogenesis. It comprises subunits S1 and S2: S1 harbors the N-terminal domain (NTD) and the receptor-binding domain (RBD) [1]. The RBD on the viral spike interacts with the angiotensin-converting enzyme 2 (ACE2) on host cells required for cell entry [2].

The neutralizing ability of antibodies targeting the S1/RBD has been reported by several studies. Neutralization is attributed to the ability of antibodies to block the S1/RBD binding to the ACE2 receptors in the respiratory tract [2]. Neutralizing antibodies (nAbs) primarily prevent viral infection by blocking the early step of infection, viral entry, and especially by interfering with virions binding to their receptor. Neutralizing antibodies can be estimated using in vitro neutralization tests, and their presence is often correlated with protective immunity [3].

In the era of COVID-19, it is important to characterize the nature of SARS-CoV2 antibodies in terms of their binding (mere mechanical blocking) and neutralizing ability (functional neutralization of virions). This characterization is necessary to assess protective antiviral immunity following infection or vaccination. The detection of the neutralizing ability of antibodies is also required to identify donors for convalescent plasma therapy [4]. The Food and Drug Administration (FDA) has recommended that convalescent plasma with a virus-neutralizing antibody titer of ≥1:160 be used for therapeutic transfusion [5].

Gomaa et al., reported that among 227 plasma samples from convalescent donors tested for nAbs against SARS-CoV-2 using a microneutralization assay, a third of the tested samples were negative for nAbs [6]. Furthermore, SARS-CoV-2 humoral immune responses have been studied extensively in a multitude of serosurveillance studies worldwide to describe pandemic dynamics, epidemiology, and associated risk factors. The epidemiological surveys primarily used anti-S tests, while a smaller percentage utilized viral neutralizing tests [7,8]. Despite the importance of nAbs in protection against SARS-CoV-2 infection, the level of nAbs has not been precisely correlated with the protection ability [9].

Several serological tests are available to determine the humoral immune response elicited by SARS-CoV-2 infection. These are based on techniques such as enzyme-linked immunosorbent assays (ELISAs), chemiluminescent immunoassays (CLIAs), and lateral flow immunoassays (LFIAs). Concerning tests for nAbs, two tests are considered gold standards. These are the plaque reduction neutralization test (PRNT) and the viral neutralization test (VNT). The PRNT can only be performed for smaller sample sizes by experienced personnel in biosafety level-3 (BSL-3) laboratories. The pseudovirus neutralization test uses a replication-deficient virus and does not require a BSL-3 laboratory, but it is more difficult to perform and is not suitable for high throughput samples. Owing to this complexity, PRNTs and VNTs are not used routinely, but they are utilized for research purposes only. Manufacturers have developed surrogate enzyme-linked immunosorbent assays (sELISAs) to overcome these drawbacks. Surrogate VNT (sVNT) tests are carried out via the competitive binding of anti-SARS-CoV-2 neutralizing antibodies with the angiotensin-converting enzyme 2 (ACE2) to block an enzyme-labeled S-RBD protein from binding its natural ligand on a microtiter plate. Surrogate ELISAs are high-throughput, cheaper, faster, and can be performed under standard laboratory safety conditions. However, there are limited data on how they compare to PRNTs and VNTs [10,11,12]. Consequently, the association between the results of tests measuring the levels of anti-S binding antibodies and those measuring the blocking neutralizing ones is gaining importance with time [13,14]. This correlation between both types of antibodies might allow the titer of anti-S antibodies to be used as an indicator, or predictor, substituting the more sophisticated neutralization tests. Several anti-S immunoassays are commercially available, but there is wide variability in their correlation with nAbs [15].

Using a commercial anti-S test which has a good correlation with nAbs and identifying a cutoff value indicating neutralization are important when it comes to predicting protective immunity. Therefore, we used two commercially available ELISA kits (anti-S and a neutralizing assay) to test their correlation and then identify an anti-S cutoff value which indicates neutralization.

## 2. Methods

### 2.1. Study Setting and Sample Size Determination

This cross-sectional study was carried out over a period of six months (January–June 2021) during the early waves of COVID-19 in Egypt. The serum samples were part of a larger survey intended to determine the seroprevalence of anti-S in the Egyptian community [16]. Based on the seroprevalence rate of SARS-CoV-2 antibodies in our previously mentioned survey (estimated to be 46.3%) [16], a minimum sample size of 388 subjects (including 155 subjects testing positive by NeutraLISA) was required to achieve a minimum power of 80% for detecting a change in the sensitivity percentage of a screening test from 0.70 to 0.80 [17], based on a target significance level of 0.05. The sample size was calculated using Power Analysis and Sample Size (PASS) 11 software.

### 2.2. Data Collection Methods and Tools

A total of 650 serum samples were tested for both SARS-CoV-2 anti-S and nAbs using kits by the same manufacturer (EUROIMMUN, Lübeck, Germany). The kits used in the study were the anti-SARS-CoV-2 Quantivac enzyme-linked immunosorbent assay (ELISA) (EUROIMMUN, Lübeck, Germany), and the SARS-CoV-2 NeutraLISA kit (EUROIMMUN, Lübeck, Germany). The first ELISA test was used to quantify immunoglobulin G (IgG) against the S1 domain of the viral spike protein of SARS-CoV-2 using a standard curve. The principle of the test depends on coating the microtiter plate with the S1 domain of the viral spike protein of SARS-CoV-2. After the addition of serum samples, bound antigen-antibody complexes were detected by the addition of another enzyme-labeled anti-human IgG (enzyme conjugate).

The second kit was used to measure the neutralizing antibodies in a competitive ELISA manner. The principle of this test depends on the coating of the microtiter plate with the S1/RBD domain of the spike antigen of SARS-CoV-2, followed by the addition of samples dissolved in ACE-2 biotinylated buffer. If nAbs are present, they compete with the binding sites of the ACE-2 for the S1/RBD receptors. The intensity of the color formed is inversely proportional to the level of antibodies in the sample [18]. Tests were performed and interpreted according to the manufacturer’s instructions.

The results of anti-S are expressed in relative unit/mL (RU/mL) and were categorized as follows: negative (<8 RU/mL), borderline (titer: 8–<11 RU/mL), and positive (titers ≥11 RU/mL). For our study, we considered borderline results to be positive. According to the manufacturer, the specificity of the QuantiVac ELISA (IgG) test was 99.8% [19].

The obtained results in RU/mL were multiplied by 3.2 and calculated in binding antibody unit/mL (BAU/mL), which is the first International Standard of the World Health Organization to detect SARS-CoV-2 neutralizing antibodies [20].

Concerning the detection of nAbs, a surrogate viral neutralization test (sVNT) with the competitive ELISA method was used (NeutraLISA, EUROIMMUN, Lübeck, Germany). This test measures the neutralizing activity of several immunoglobulins, which are Igs A, M, and G [18]. According to EUROIMMUN, this kit has a specificity of 99.7% and its sensitivity is 95.9% [21]. As instructed, the percentage inhibition (%IH) results were interpreted as follows: % IH < 20 were considered “negative”, % IH ≥ 20 to <35 were considered “borderline”, while % IH ≥ 35 indicated a “positive” result [18]. Similar to our approach with borderline results for anti-S, borderline results for nAbs were also considered positive.

Tests of association and quantitative correlation were performed between both antibodies. After ensuring a good correlation existed between both tests, a receiver operating characteristic (ROC) curve analysis was constructed to generate a cutoff point for anti-S above which the NeutraLISA test was predicted to be positive at an optimum sensitivity and specificity.

### 2.3. Statistical Analysis

Statistical analysis was performed using SPSS-V25 (IBM Corp., Armonk, NY, USA) and Medcalc. Concordance analyses (Cohen’s Kappa) were performed to compare the results of each antibody assay and the results were interpreted as follows: value ≤ 0 as no agreement, 0.01–0.20 as none to slight, 0.21–0.40 as fair, 0.41–0.60 as moderate, 0.61–0.80 as substantial, and 0.81–1.00 as almost perfect agreement [22]. Receiver operating characteristic (ROC) curve analysis was used to identify optimized thresholds (cutoff indices). Means were given with standard deviation (SD) and medians with interquartile range (IQR). For comparison of means, the Mann–Whitney U and Kruskal–Wallis tests were used. The Spearman’s correlation coefficient was determined for the linear correlation of two variables; *p*-values  <  0.05 were considered significant differences.

## 3. Results

A total of 650 serum samples were tested for both anti-spike and neutralizing antibodies against SARS-CoV-2 using kits by the same manufacturer (EUROIMMUN, Germany). Borderline results constituted 10.5% (68 samples) of all nAb results, and 2.9% (19 samples) of all anti-S results. Borderline results were considered positive for both antibodies to avoid confusion in statistical analyses.

Anti-S positivity was prevalent in 68.9% of samples (n = 448), with a median (IQR) titer of 44.0 (77.48) RU/mL in positive samples (equivalent to 140.8 BAU/mL) and a median (IQR) of 20.40 (60.53) (equivalent to 65.3 BAU/mL) in total samples. The presence of nAbs was prevalent in 53.8% of samples (n = 350), with a median (IQR) IH% of 62.18 (46.29) in positive samples and median (IQR) of 24.81(59.18) in total samples (Table 1).

**Figure 1:**
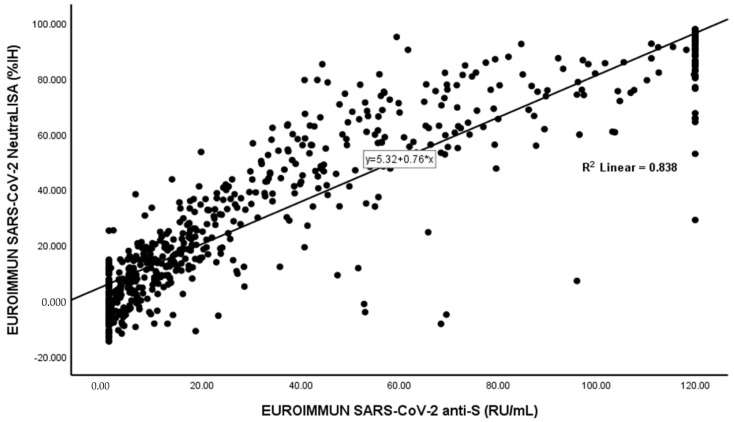
Correlation of EUROIMMUN SARS-CoV-2 anti-S (RU/mL) with EUROIMMUN SARS-CoV-2 NeutraLISA (%IH)* (n = 650).

There was a significant correlation between levels of anti-S and nAbs (Spearman’s rho = 0.913, *p*-value < 0.01) (Figure 1).

Among the positive samples for anti-S, 77.0% of them (n = 345) were also positive for nAbs, while 23.0% were negative. Among the negative samples for anti-S, the majority were also negative for nAbs, while five samples (2.5%) had a neutralizing activity despite being negative for anti-S. There was a substantial agreement between anti-S and nAbs (Cohen’s kappa coefficient (κ) = 0.658 (0.602–0.714), SE = 0.029, *p*-value < 0.01), with an overall percent agreement of 83.38%. Considering NeutraLISA as a gold standard, anti-S was found to have a sensitivity of 98.57%, specificity of 65.66%, NPV of 97.5%, and PPV of 77.0% (Table 2). 

**Figure 2:**
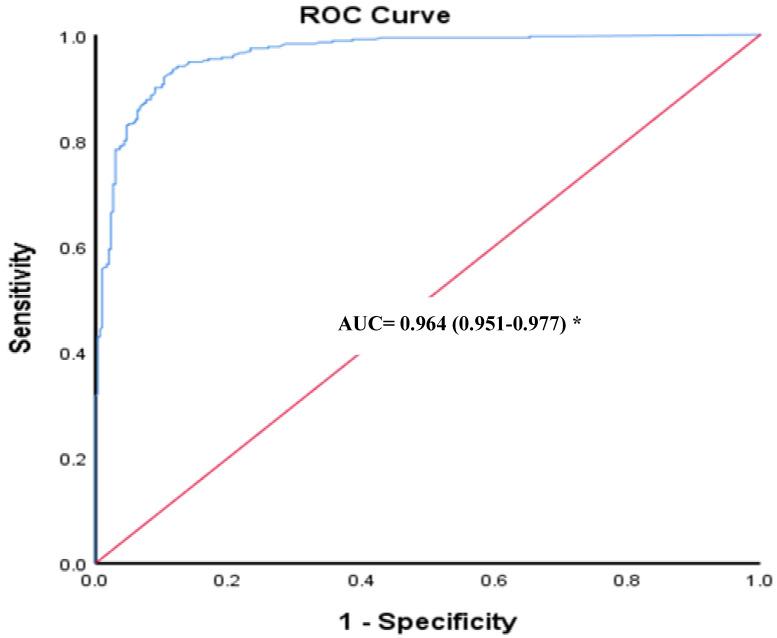
A ROC analysis illustrating the optimized cutoff value for the anti-spike immunoglobulin, in relation to the prevalence of nabs (AUC= 0.964 (0.951–0.977). * *p*-value < 0.01.

The ROC curve showed a good predictive value in terms of the anti-S titers for the prevalence of nAbs (area under the curve = 0.964 (0.951–0.977). When the anti-S titer exceeded a value of 18.1 RU/mL (57.9 BAU/mL), nAbs were also positive at a sensitivity of 90.0% [95% CI: 68.6–89.6%] and a specificity of 91% [95% CI: 73.5–97.9%] (Figure 2). Lower anti-S titers exhibited higher diagnostic sensitivities but lower specificities and vice versa. For instance, when the anti-S titer was 10 RU/mL, the prediction of nAbs prevalence had a sensitivity of 97.7% and a specificity of 73%. Meanwhile, when the anti-S titer was 40 RU/mL, the prediction of nAbs prevalence had a sensitivity of 67.1% and a specificity of 98%.

## 4. Discussion

To the best of the authors’ knowledge, there are no current data available regarding the titer of neutralizing antibodies that protect persons from SARS-CoV-2 infection. However, the role of nAbs as a marker of immunity against COVID-19 infection is proven. Lucas et al. reported that the early appearance of nAbs correlates with better COVID-19 clinical trajectory and prognosis among hospitalized patients. They also found that patients who had lower SARS-CoV-2 viral loads in their nasal swabs had higher serum nAbs [23]. Similar protective effects of nAbs were reported in studies on vaccine recipients and donors of convalescent plasma [3,6]. Cromer et al. reported that a decline in neutralizing antibodies against SARS-CoV-2 occurs in convalescent patients, resulting in reinfection [24]. This highlights the importance of laboratory tests for nAbs to evaluate the immune status of such individuals.

An International Standard (IS) containing a known amount of anti-SARS-CoV-2 immunoglobulins with well-characterized neutralizing activity was made available by the WHO in December 2020 to allow for the accurate calibration of assays to the international unit (IU) for neutralizing antibodies or binding antibody units (BAU) for binding antibodies, thereby reducing interlaboratory variation and creating a common language for reporting data [20]. This standardization of measuring units is encouraged among kit manufacturers and vaccine developers to allow for the comparison of results from studies on infection or post-vaccination and to precisely identify correlates of protection [25]. Regarding the tests used in our study, the Quantivac SARS-CoV-2 test has an established conversion factor (as described by the manufacturer), where results in RU/mL are multiplied by a factor of 3.2 to obtain results expressed in BAU/mL, as recommended. However, no such conversion factor is available for the NeutraLISA kit [26]. Nevertheless, since our work was not aiming to identify a correlate of protection against infection and we aimed to merely identify a positivity cutoff for neutralizing antibodies, the lack of a conversion factor did not affect the rationale of our study or the reliability of our results.

Our study used a surrogate VNT (sVNT) rather than the conventional pseudovirus-based SARS-CoV-2 neutralizing antibody assay. This sVNT test (SARS-CoV-2 NeutraLisa test) has the advantage of being easy to perform, not requiring a BSL-3 laboratory and the production of rapid results. The sVNT is also suitable for high-throughput testing and/or fully automated testing after minimal adaptation. According to Tan et al., there was a good correlation between the results of the RBD ELISA and the RBD sVNT [27]. 

We chose the EUROIMMUN NeutraLISA test and the anti-S test Quantivac by the same manufacturer to maximize the accuracy of our results and reduce variation owing to differences in manufacturing techniques between different commercial brands. Montesinos et al. compared the results of six binding assays and compared their clinical performances against a plaque reduction neutralization test (PRNT) at a 1/80 titer and concluded that EUROIMMUN QuantiVac IgG showed a better ability to detect nAbs than four other assays with an area under the ROC curve of 0.95 (95% CI: 0.92–0.98) [28]. Pieri et al. also reported an overall agreement of 95.45% and a correlation of r = 0.810 between the EUROIMMUN sVNT and the results of the gold standard VNT [26]. Overall, these results encouraged us to choose the EUROIMMUN sVNT in our study for cutoff determination.

In our study, among the positive samples for anti-S, 77.0% (n = 345) were positive for nAbs. This indicates that the humoral response against S-protein for the most part also involves the production of neutralizing antibodies. Several studies reported such findings [4,29,30]. Almost a quarter of the sera (23.0%) had no nAbs despite being positive for anti-S. Researchers have explained this by the fact that SARS-CoV-2 infection/vaccination may often fail to induce sufficient B-cell expansion and maturation to generate high-titer neutralizing antibodies [31].

Considering NeutraLISA as a gold standard, anti-S was found to have a sensitivity 98.57%, a specificity 65.66%, an NPV of 97.5%, and a PPV of 77.0%, with an AUC of 0.964 (0.951–0.977). There was a significant correlation between levels of anti-S and nAbs (Spearman’s rho= 0.913, *p*-value < 0.01) and a substantial agreement between both tests (Cohen’s kappa coefficient = 0.658 (0.602–0.714), *p*-value < 0.01), with an overall percentage agreement of 83.38%. The manufacturer (EUROIMMUN) tested 111 samples with anti-SARS-CoV-2 Quantivac (IgG) and NeutraLISA kits and stated a qualitative agreement of 99.1% (borderline results were excluded) [21]. In our case, the agreement between both tests was lower than that reported by the manufacturer, and this could be a result of our inclusion of borderline results with positive ones. The agreement between anti-S and nAbs denotes that the humoral response against S-protein also primarily involves the production of neutralizing antibodies [10]. Similar to our work, Dinç et al. reported a correlation between both kits by EUROIMMUN (rs = 0.496, *p* < 0.001) [32].

Valdivia et al., reported a strong correlation between SARS-CoV-2 anti-S and nAbs. However, their study used the EUROIMMUN SARS-CoV-2 IgG ELISA (rho  =  0.73) and a pseudotyped virus assay for 50% virus neutralization [33].

When the spike antibodies had a titer exceeding 18.1 RU/mL (57.9 BAU/mL), nAbs were also positive, with a sensitivity of 90.0% [95% CI: 68.6–89.6%] and specificity of 91% [95% CI: 73.5–97.9%]. A study reported a similar approach, where a cutoff anti-RBD IgG antibody level of 9.95 S/CO resulted in a sensitivity of 84.0% and a specificity of 86.3% for the prediction of seropositivity for neutralizing antibodies [34]. Another study evaluated the antibody response (both spike and neutralizing antibodies) among vaccinated participants using the same EUROIMMUN kits used in the present study. In that study, the anti-S test was reported to be a reliable predictor among cases with positive neutralizing antibodies (area under the curve = 0.929). In that same study, when the anti-S titer exceeded 40 RU/mL (128 BAU/mL), neutralization was also positive, with a sensitivity of 80.6% (95% CI: 68.6–89.6%) and specificity of 90% (95% CI:73.5–97.9%) [35]. In that study, borderline results were considered to be negative, while in the present study, borderline results (10.5%) were considered to be positive, and this might explain the relatively higher cutoff chosen by their study compared to ours. Moreover, our larger sample size (n = 650) compared to theirs (n = 92) might also contribute to the better accuracy and lower cutoff value.

Generally, to determine an optimized predictive cutoff out of a ROC analysis, this requires knowing the purpose of the test. In the context of the COVID-19 pandemic, if an immunoassay is required as a diagnostic aid, such as in serosurveillance studies, then a high sensitivity is preferred (exceeding 90%) over a high specificity. However, if antibody tests were required for the release of patients from quarantine, then specificity would be more important than sensitivity. This is because a low specificity could lead to infectious patients being released from the quarantine to the community, leading to the perpetuation of the infection in the community [36]. 

In our study, at lower anti-S titers, e.g., 10 RU/mL, the prediction of nAbs positivity had a sensitivity of 97.7% and a specificity of 73%. Meanwhile, when the anti-S titer was 40 RU/mL, the prediction of nAbs positivity had a sensitivity of 67.1% and a specificity of 98%. It is noteworthy to observe the variation in sensitivity and specificity at the titer 40 RU/mL between our study and the other study described earlier [35], as we recorded a lower sensitivity but a higher specificity at that titer. Again, our larger sample size and discrepancy in terms of the inclusion of borderline results might have contributed to these variations in diagnostic performance, even though both studies used the same commercial kits for both antibodies. 

In the present study, five samples (2.5%) had a neutralizing activity despite being negative for anti-S. This might be explained by the fact that, although the RBD specifically is the main target for nAbs, other areas/epitopes of S protein may also elicit nAbs [29]. Possible reasons for this include the fact that the NeutraLISA test, as described by its manufacturer, does not only measure only neutralizing IgG but also neutralizing activity by IgA and IgM. On the other hand, the Quantivac kit, as stated by the same manufacturer, measures the anti-S binding ability of IgG only. EUROIMMUN recommends repeating the investigations 7 to 14 days later to overcome this discrepancy [19]. Marot et al. reported that in their convalescent plasma samples, the purified IgA had a greater neutralizing capacity than IgG at earlier time points (2 weeks post-infection until 3 months), whereas the opposite pattern was observed after 3 months [37].

It is noteworthy to mention that the samples of our study were collected near the end of the second wave of the pandemic and throughout the whole third wave (January–June 2021). During this time, the dominant viral variants belonged to the Pangolin lineage C.36 (especially C.36.3) [38]. According to official national reports in August 2021, at that time, the Delta variant had not yet been detected in Egypt, despite being discovered in several other countries [39]. Delta (B.1.617.2) and Omicron (B.1.1.529) variants are known for their immunity evasion and escape from neutralization owing to mutations in their N-terminal domain of the spike protein, which is the primary neutralizing epitope [40]. Ai et al. reported that their pseudovirus assay demonstrated that Omicron virus neutralization by plasma of convalescent or twice-vaccinated people (with BNT162b2, mRNA-1273, ChAdOx1, Ad26.COV2.S, Sputnik V, BBIBP-CorV) was significantly reduced or absent in comparison with the ancestral strain [41]. Studies on viral neutralization should always be interpreted in the context of circulating viral variants in a specific geographical location.

### Strengths and Limitations

Our study included a relatively large number of samples, which provided more reliable results when the cutoff was calculated. Although several studies utilized the Quantivac (EUORIMMUN) kit, there is a scarcity in terms of the publications available on the sVNT NeutraLISA (EUORIMMUN) test. To the best of our knowledge, no other study has been published that predicted a cutoff value for this anti-S Quantivac test using the NeutraLISA kit.

The limitations of the study include the absence of a conversion factor for the NeutraLISA test in terms of the WHO international standards (IU/mL). This might have had helped in identifying the values of nAbs that exceed the cutoff of anti-S in units that are standardized between different neutralization tests.

## 5. Conclusions

Our results indicate that the anti-SARS-CoV-2 QuantiVac ELISA IgG test (EUROIMMUN, Lübeck, Germany) can be correlated with the neutralizing antibody response against SARS-CoV-2. This can be helpful following natural SARS-CoV-2 infection and vaccination or for the identification of convalescent plasma donors.

## Figures and Tables

**Table 1:** Distribution of the 650 samples according to their median (IQR) results for SARS-CoV-2 anti-S IgG titer (RU/mL) and SARS-CoV-2 nAbs (%IH).

		No.	%	Median: Titer (anti-S)/IH%(nAbs)	IQR
**SARS-CoV-2 anti-S IgG**	Negative	202	31.1	1.65	4.10
	Positive	448	68.9	44.00	77.48
	Total	650	100.0	20.40	60.53
**SARS-CoV-2 nAbs**	Negative	300	46.2	4.82	15.46
	Positive	350	53.8	62.18	46.29
	Total	650	100.0	24.81	59.18

**Table 2:** Agreement and performance of EUROIMMUN SARS-CoV-2 anti-S with EUROIMMUN SARS-CoV-2 NeutraLISA as a gold standard (n = 650).

		EUROIMMUN SARS-CoV-2 NeutraLISA		Total
		Negative (n = 300)	Positive (n = 350)	
**EUROIMMUN SARS-CoV-2 anti-S**	**Negative**	197 (97.5%)	5 (2.5%)	202
	**Positive**	103 (23.0%)	345 (77.0%)	448
Cohen’s kappa coefficient (κ) = 0.658 (0.602–0.714), SE = 0.029, *p*-value < 0.01 Overall percent agreement = 83.38%				
Sensitivity = 98.57% (96.69–99.53%)				
Specificity = 65.66% (59.99–71.02%)				
* NPV = 97.52% (94.26–98.95%)				
** PPV = 77.00% (74.11–79.67%)				
*** Positive LR = 2.87 (2.45–3.35)				
**** Negative LR = 0.02 (0.00–0.05)				

* NPV: negative predictive value; ** PPV: positive predictive value; *** Positive LR: positive likelihood ratio; **** Negative LR: negative likelihood ratio.

## Data Availability

Not applicable.
